# *Helicobacter ailurogastricus* in Patient with Multiple Refractory Gastric Ulcers, Japan

**DOI:** 10.3201/eid2904.221807

**Published:** 2023-04

**Authors:** Masaya Sano, Emiko Rimbara, Masato Suzuki, Hidenori Matsui, Miwa Hirai, Sae Aoki, Tsuyoshi Kenri, Keigo Shibayama, Hidekazu Suzuki

**Affiliations:** Tokai University School of Medicine, Kanagawa, Japan (M. Sano, M. Hirai, H. Suzuki);; National Institute of Infectious Diseases, Tokyo, Japan (E. Rimbara, M. Suzuki, H. Matsui, S. Aoki, T. Kenri);; Nagoya University Graduate School of Medicine, Aichi, Japan (K. Shibayama)

**Keywords:** *Helicobacter ailurogastricus*, *Helicobacter heilmannii*, non-*Helicobacter pylori Helicobacter*, gastric ulcers, bacteria, Japan

## Abstract

We report the isolation of *Helicobacter ailurogastricus*, a *Helicobacter* species that infects cats and dogs, from a person with multiple refractory gastric ulcers. In addition to *H. suis*, which infects pigs, *Helicobacter* species that infect cats and dogs should be considered as potential gastric pathogens in humans.

A 61-year-old man in Japan had multiple ulcers diagnosed on esophagogastroduodenoscopy (EGD) performed during his annual health checkup and was referred to Tokai University Hospital (Kanagawa, Japan) because of an inadequate therapeutic response. Histologic examination of tissue from the ulcer site showed inflammatory cells and few findings suggestive of malignancy. Hematoxylin and eosin staining showed spiral bacteria resembling a *Helicobacter* species.

Test results for *H. pylori* serum antibodies and stool antigen were negative. The patient had onset of epigastric discomfort after his work became busy but attributed his symptoms to his work burden and did not seek medical care. Although he had not taken nonsteroidal antiinflammatory drugs or aspirin, he did not respond to therapy, even with the administration of the antisecretory agent vonoprazan (20 mg), and had multiple refractory gastric ulcers diagnosed.

After obtaining informed consent, we enrolled the patient in a clinical trial investigating the effects of non–*H. pylori Helicobacter* (NHPH) infections on intractable ulcers and gastric mucosa–associated lymphoid tissue lymphoma. On August 24, 2021, we assessed the patient for NHPH by using culture and PCR of gastric biopsy samples collected during EGD (Appendix). EGD showed no atrophy in the background gastric mucosa, healing of the ulcers observed previously, multiple erosions, and residual ulcers in the antrum (Figure, panel A). The PCR test result for NHPH was positive, but the bacterial culture result was negative. On November 30, 2021, a repeat EGD to assess ulcer healing status showed further healing. Repeat culture and PCR tests for NHPH were both positive. We isolated *Helicobacter* spp. strain NHP21-4376 from the greater curvature of the gastric antrum and NHP21-4377 from the lesser curvature.

**Figure:**
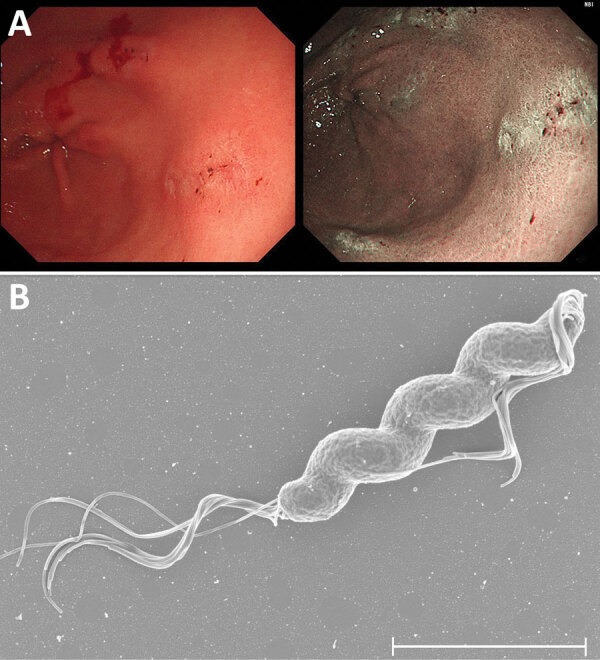
Endoscopic images from a gastric ulcer patient infected with *Helicobacter ailurogastricus* and morphologic observation and genomic comparison of isolated *H. ailurogastricus* NHP21-4376 and NHP21-4377 strains, Japan. A) Multiple linear erosions and small ulcers on the background mucosa with no evidence of atrophy in the gastric antral area. B) Scanning electron micrograph of *Helicobacter ailurogastricus* strain NHP21-4377. Scale bar indicates 2 μm.

The microorganisms had a corkscrew-like spiral form (Figure, panel B) resembling that of *Helicobacter suis*, the most prevalent NHPH species in the human stomach. We performed whole-genome sequencing of the NHP21-4376 and NHP21-4377 strains by using the Illumina platform (Illumina, https://www.illumina.com) (Appendix). We assembled the Illumina reads de novo by using Shovill 1.1.0 (https://github.com/tseemann/shovill) with the default parameters. We determined the bacterial species by calculating the average nucleotide identity (ANI) using pyani 0.2.12 (https://github.com/widdowquinn/pyani). Strains NHP21-4376 and NHP21-4377 had >98% identity with *H. ailurogastricus* strains, including the type strain ASB7^T^, indicating that they were *H. ailurogastricus* (Appendix Figure 1). 

Phylogenetic analysis based on 342 core genes among gastric *Helicobacter* species also confirmed that NHP21-4376 and NHP21-4377 are in the same clade as *H. ailurogastricus* strains ASB7^T^ and ASB9 and are distinct from *H. suis* strains (Appendix Figure 2). We deposited draft genome sequences of *H. ailurogastricus* into GenBank (NHP21-4376 accession nos. BSCV01000001–64 and NHP21-4377 accession nos. BSCW01000001–66). 

Antimicrobial susceptibility tests showed that *H. ailurogastricus* NHP21-4376 had a high MIC for levofloxacin (Table) and that the NHP21-4376 strain had a Ser to Arg mutation at position 78 in the quinolone resistance–determining region of DNA gyrase A (Appendix Figure 3). This position corresponds to Asn at position 87, where its mutation is responsible for fluoroquinolone resistance in *H. pylori* (*1*).

**Table:** Antimicrobial susceptibilities of *Helicobacter ailurogastricus* strains ASB7^T^ and NHP21-4376 isolated from patient, Japan, 2021

Strain	Host	MIC, mg/L					
		Amoxicillin	Clarithromycin	Metronidazole	Minocycline	Gentamicin	Levofloxacin
ASB7^T^	Cat	0.25	<0.25	16	<2	<4	<0.5
NHP21-4376	Human	1	<0.25	16	<2	<4	4

*H. suis*, which is the most prevalent NHPH species in humans, is believed to originate in pigs. Virulence-associated features were recently shown in *H. suis* isolates obtained from human stomachs (*2*); gastric ulcer recurrence was not observed in the patient infected with *H. suis* after *H. suis* eradication (*2*). Furthermore, *H. ailurogastricus* and *H. heilmannii* are 2 of the most prevalent NHPH strains infecting the human stomach, after *H. suis* (*3*,*4*). *H. ailurogastricus* was formerly classified as *H. heilmannii.* *H. heilmannii* and *H. ailurogastricus* are prevalent *Helicobacter* species that infect the stomachs of cats (*5*). Moreover, *H. ailurogastricus* is shown to be the prevalent gastric *Helicobacter* species infecting the stomach of cats and dogs in Japan (Appendix Table, Figure 4). 

In this case, the patient was strongly suspected to have acquired the infection from his cats, although the stool of his pets could not be analyzed because the patient’s consent was not obtained. The patient has not had a recurrence of multiple ulcers but remains positive for *H. ailurogastricus*. The limitation of this case report is that, although we succeeded in culturing *H. ailurogastricus* in the stomach of this patient and the drug-susceptibility test has determined the regimen for eradication, we have not yet been able to perform eradication therapy. Therefore, the efficacy of eradication in *H. ailurogastricus* infections has not been confirmed. *H. ailurogastricus* eradication therapy will be administered at the next patient visit to prevent ulcer recurrence. 

The clinical importance of NHPH infection in the human stomach has been increasing in the post–*H. pylori* era. Because NHPH species such as *H. suis* and *H. ailurogastricus* cannot be detected by most *H. pylori* diagnostic tests, such as the urea breath test and stool antigen test, NHPH infections should be considered when routine *H. pylori* tests are negative, despite the presence of inflammatory findings in the gastric mucosa.

Appendix Additional information about *Helicobacter ailurogastricus* in a patient with multiple refractory gastric ulcers, Japan.
