# Supplementary material for: Helicobacter ailurogastricus in Patient with Multiple Refractory Gastric Ulcers, Japan
Source: Emerg Infect Dis. 2023 Apr;29(4):833–5. doi: 10.3201/eid2904.221807 (PMC10045715; doi:10.3201/eid2904.221807)
Supplement: Appendix — Additional information about Helicobacter ailurogastricus in a patient with multiple refractory gastric ulcers, Japan. [file 22-1807-Techapp-s1.pdf]

Article DOI: https://doi.org/10.3201/eid2904.221807

Helicobacter ailurogastricus in a Patient with Multiple Refractory Gastric Ulcers, Japan

Appendix

Methods

Non-Helicobacter pylori Helicobacter (NHPH) testing

To detect NHPH infections by PCR, DNA was extracted from the homogenates of gastric biopsy specimens using DNeasy Blood & Tissue Kits (Qiagen, Hilden, Germany). Then, the DNA was used as a template for probe-based real-time PCR targeting the NHPH-specific region of the 16S rRNA gene. The sequences of the two sets of primers and probes were as follows: NHPH_16S_F (5′-CAAGTCGAACGATGAAGCCTA-3′), NHPH_16S_R (5′-ATTTGGTATTAATCACCATTTCTAGT-3′), and NHPH_16S_probe (5′-/56-FAM/TTACTCACC/ZEN/CGTGCGCCACTAATC/3IABkFQ/-3′) for targeting the NHPH 16S rRNA gene. To detect NHPH infections by culture, the method for *Helicobacter suis* isolation from human gastric biopsies, as described in a previous study (*1*), was used. Briefly, the gastric biopsy specimen was homogenized in *Brucella* broth (Difco Laboratories, Detroit, MI, USA) adjusted to pH 5.0 using hydrochloric acid. The tissue homogenates were inoculated onto NHPH agar plates containing 1.5% (w/v) agar, Brucella broth, 20% (v/v) heat-inactivated fetal bovine serum, *Campylobacter*-selective supplement (Skirrow; Oxoid, Basingstoke, UK), Vitox

supplement (Oxoid), and hydrochloric acid to adjust the pH to 5.0 and incubated for more than 7 days in a humidified gas mixture (5% O_2, 12% CO_2, and 83% N_2) at 37°C. The grown colonies of the primary culture were inoculated onto NHPH agar plates and enriched by modified biphasic culture for 120 h, with shaking in a humidified gas mixture at 37°C.

Genomic Methods

Whole-genome sequencing of the *Helicobacter* spp. strains was performed using MiniSeq (Illumina, San Diego, CA, USA). The library for Illumina sequencing (150-bp paired-end; insert size, 500–900 bp) was prepared using a Nextera XT DNA Library Prep Kit. The Illumina reads were assembled de novo using Shovill v1.1.0. (https://github.com/tseemann/shovill) with the default parameters to acquire draft genome sequences. Core genome alignments among *Helicobacter* strains were determined using Roary version 3.13.0 (https://github.com/sanger-pathogens/Roary) with the default parameters. Maximum-likelihood phylogenetic trees were constructed using RAxML-NG v. 1.1 (https://github.com/amkozlov/raxml-ng) with core gene alignments. Bacterial species were determined by calculating the average nucleotide identity (ANI) using pyani 0.2.12 (https://github.com/widdowquinn/pyani).

References

1. Rimbara E, Suzuki M, Matsui H, Nakamura M, Morimoto M, Sasakawa C, et al. Isolation and characterization of *Helicobacter suis* from human stomach. Proc Natl Acad Sci U S A. 2021;118:e2026337118. PubMed https://doi.org/10.1073/pnas.2026337118

2. Kubota-Aizawa S, Ohno K, Fukushima K, Kanemoto H, Nakashima K, Uchida K, et al. Epidemiological study of gastric *Helicobacter* spp. in dogs with gastrointestinal disease in Japan

and diversity of *Helicobacter heilmannii* sensu stricto. Vet J. 2017;225:56–62. PubMed https://doi.org/10.1016/j.tvjl.2017.04.004

3. Kubota-Aizawa S, Ohno K, Kanemoto H, Nakashima K, Fukushima K, Uchida K, et al. Epidemiological study on feline gastric *Helicobacter* spp. in Japan. J Vet Med Sci. 2017;79:876–80. PubMed https://doi.org/10.1292/jvms.16-0567

Appendix Table. Prevalence of gastric *Helicobacter* species among dogs and cats in Japan*

	No. (%) of strains					
Species	Dog (n = 47)		Cat (n = 24)		Total (n = 71)	
H. ailurogastricus	1	(2.1)	6	(25.0)	7	(9.9)
H. heilmannii	1	(2.1)	0	(0)	1	(1.4)
H. bizzozeronii	1	(2.1)	2	(8.3)	3	(4.2)
H. felis	1	(2.1)	4	(16.7)	5	(7.0)
H. pylori	1	(2.1)	0	(0)	1	(1.4)
Not classified	42	(89.4)	12	(50.0)	54	(76.1)

*The prevalence was estimated from the sequences obtained from gastric specimens of dogs (*2*) and cats (*3*) in Japan.

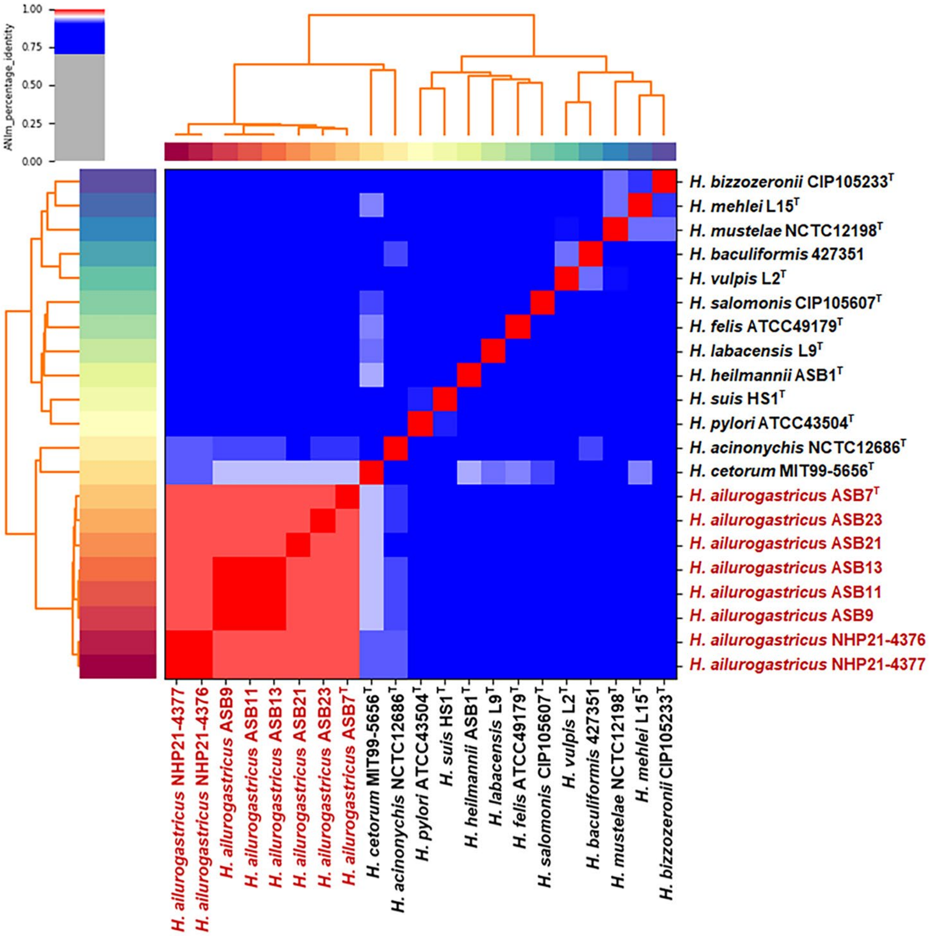

Appendix Figure 1. Average nucleotide identity among gastric *Helicobacter* species. ANI was calculated via pyani 0.2.12 using the ANI MUMmer/NUCmer method. Strains denoted in red are *H. ailurogastricus* including the NHP21–4376 and NHP21–4377 strains isolated in the study.

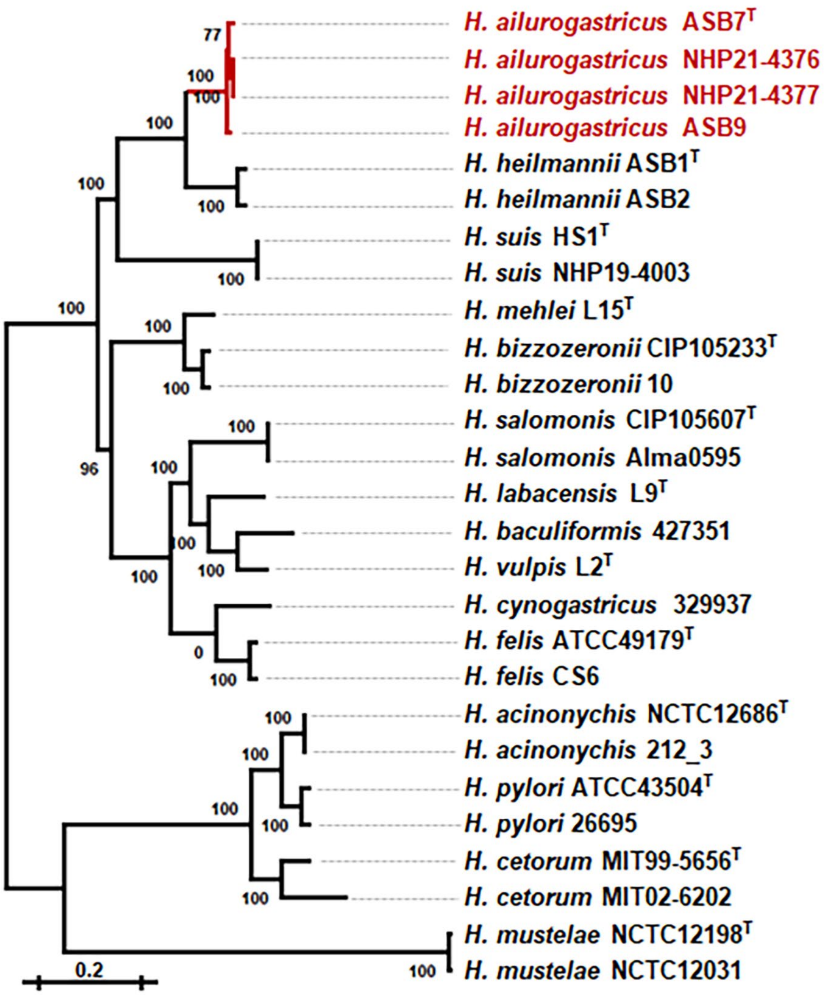

Appendix Figure 2. Phylogenetic tree based on 342 core genes among gastric *Helicobacter* species. Core gene alignment was constructed using Roary version 3.13.0, and the phylogenetic tree was constructed using RAxML-NG v. 1.1. The scale bar indicates the number of base substitutions per site. The lines indicate *Helicobacter ailurogastricus* strains including NHP21–4376 and NHP21–4377 obtained in this study.

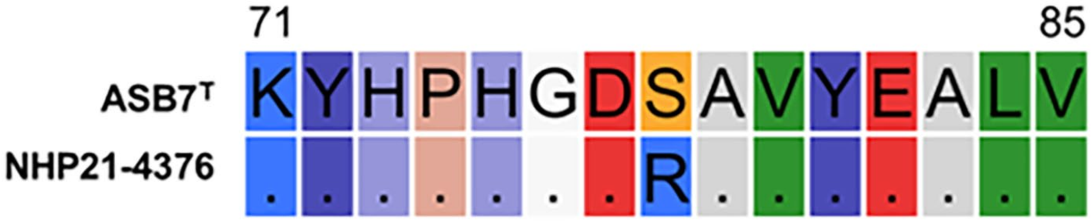

Appendix Figure 3. Quinolone resistance-determining region in DNA gyrase A of *Helicobacter ailurogastricus* strains ASB7^{T} from a cat and NHP21–4376 from a human patient.

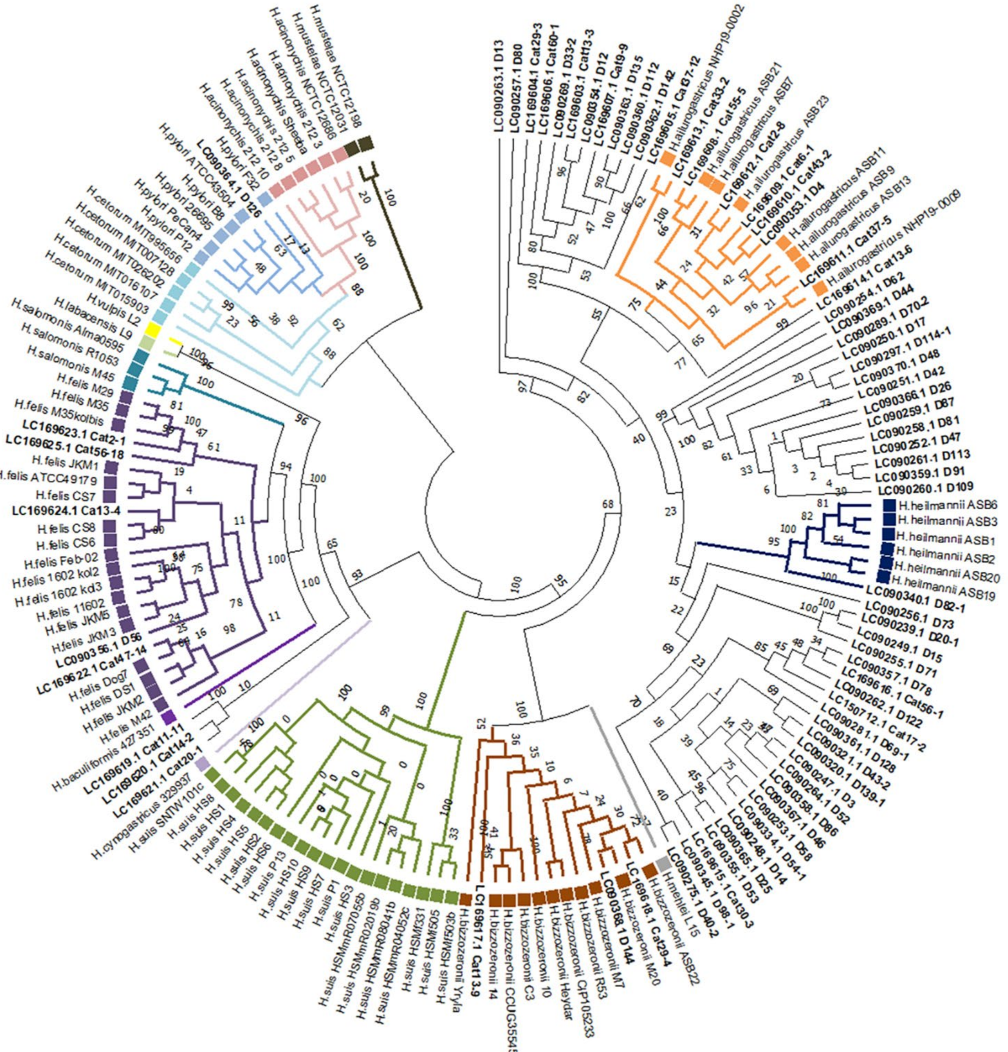

Appendix Figure 4. Phylogenetic tree generated from *ureAB* gene sequences of gastric *Helicobacter* species. The phylogenetic tree generated from the 161 *ureAB* gene sequences. The sequences were aligned by MAFFT version 7.49 and the tree was constructed using RAxML-NG version 1.1.0 with a GTR+G+I model and 100 bootstrap replicates. Numbers indicate bootstrap percentages, and the scale bar indicates the number of base substitutions per site. The *ureAB* sequences of the reference strains (squares) were extracted from whole genome sequences of gastric *Helicobacter* species obtained from NCBI. The *ureAB* sequences shown as the GenBank no. (LCXXXXXX) are obtained from cats and dogs in Japan.
